# Pregabalin Abuse in Combination With Other Drugs: Monitoring Among Methadone Patients

**DOI:** 10.3389/fpsyt.2019.01022

**Published:** 2020-02-11

**Authors:** Massimo Lancia, Angela Gambelunghe, Alessio Gili, Mauro Bacci, Kyriaki Aroni, Cristiana Gambelunghe

**Affiliations:** ^1^ Forensic and Sports Medicine Section, Department of Surgery and Biomedical Science, University of Perugia, Perugia, Italy; ^2^ Occupational Medicine, Respiratory Diseases and Toxicology Section, Department of Medicine, University of Perugia, Perugia, Italy; ^3^ Hygiene and Public Health Section, Department of Experimental Medicine, University of Perugia, Perugia, Italy

**Keywords:** dual diagnosis, hair analysis, poly-drug abuse, pregabalin, pregabalin abuse

## Abstract

**Introduction:**

In recent years, there has been an increase in the non-medical use of psychoactive prescription drugs including pregabalin (PGB). Studies have shown that multiple drug users and patients in methadone treatment programs administered PGB at high dosages in order to achieve euphoria, reduce withdrawal symptoms, or potentiate the effects of methadone. For these reasons, accurate toxicological monitoring is required for these high-risk individuals.

**Materials and Methods:**

The present study investigated whether PGB could be detected in the hair samples of 250 patients with a history of opiate dependency, and under toxicological surveillance assess their compliance with methadone maintenance therapy.

**Results:**

Opiates were found in 54/250 of all hair samples, while cannabis was present in 74/250 patients, cocaine was detected in 21/250 patients, and benzodiazepines without prescription were identified in 49/250 patients. As expected, methadone was present in all 250 patients (100%). PGB without prescription was found in the hair samples of 35/250 patients (14%). Of these, 91.43% were male, 48.57% were <30 y old, and 45.71% were between ages 30 and 50 y. There were no apparent associations among PGB use, daily methadone dosage, and duration of methadone maintenance therapy. Psychiatric comorbidities were present in 25.71% of the patients abusing PGB. Anxiety (55.56%) and depression (33.33%) were the most prevalent psychiatric disorders.

**Discussion:**

Most of the patients taking PGB (57.14%) used other drugs (especially opiates) concurrently. The utility of hair analysis is explained by easy and rapid sample collection and the ability of the hair to reflect long-term drug use and incorporate drug metabolites. The findings of this study suggested that PGB has significant potential for abuse by high-risk populations such as opioid users and patients with dual diagnosis. These risks are particularly high in cases of poly-drug use and drug intake that are not in compliance with prescription guidelines.

## Introduction

Non-medical prescription drug use is a widely growing concern with severe consequences. According to the 2015 National Survey on Drug Use and Health in the United States, prescription psychotherapeutic drugs ranked second only to cannabis as the most prevalent illicit drug use category across all age groups (1). Similar figures were reported for European countries (2).

Sedatives and hypnotics are commonly misused prescription drugs. These include barbiturates, benzodiazepines (BZDs) and BZD-like drugs such as z-hypnotics, opioids, and opioid substitution medications which are used for pain relief, and stimulants to treat attention deficit and hyperactivity disorder (ADHD) (3). Other medications such as antidepressants, anti-Parkinson drugs, cough and cold medicines, and stimulants such as methylphenidate are also misused (2, 4, 5).

Non-medical prescription drug use typically involves taking medication not prescribed for the user or consuming it in a manner not intended by the prescriber. Examples include taking doses higher than those recommended and using non-approved administration routes. These substances are often consumed in the context of poly-drug use (6, 7).

### Pregabalin General Pharmacology

Pregabalin (PGB) [(S)-3-(aminomethyl)-5-methylhexanoic acid] is a centrally-acting neuromodulating agent, and has been in the U.S. market since 2005 (8). As a relatively more powerful analog of gabapentin, it is used to treat epilepsy/partial seizures, neuropathic pain including diabetic peripheral neuropathy, post-herpetic neuralgia, and fibromyalgia. In Europe, PGB is approved for the management of generalized anxiety disorder (GAD) (9). PGB has been extensively prescribed off-label for psychiatric conditions such as bipolar disorder, alcohol/narcotic withdrawal states, and ADHD (10).

Though it is a gamma-aminobutyric acid (GABA) analog, PGB does not bind GABAA, GABAB, or benzodiazepine receptors. Moreover, serum GABA levels do not change in response to short-term PGB administration. PGB has a high affinity for the α-2-δ (alpha-2-delta) subunit of voltage-gated calcium channels. Thus, calcium influx is reduced following PGB intake (8) and the calcium-dependent release of neurotransmitters involved in pain pathways (substance P, glutamate, and noradrenaline) decreases (8, 11, 12).

PGB has predictable pharmacokinetics. It has greater than 90% oral bioavailability regardless of dose or frequency of administration. Up to 98% of it is excreted unchanged through the kidneys (13). It has no reported pharmacokinetic drug-drug interactions and minimally binds to serum proteins. Plasma PGB concentrations peak within 1.5 h of administration. PGB readily crosses the blood-brain barrier and has comparatively fewer side effects (13). The foregoing pharmacological and pharmacokinetic properties of PGB make it an excellent choice for clinical use. It is widely prescribed for all of the aforementioned medical conditions. According to Pharma Marketing (2018), PGB reached the tenth position in terms of gross sales in 2017 (~5.1 billion USD) and its annual sales growth rate was ~2.8% (7).

### Pregabalin Abuse Potential

The first reports of PGB abuse and dependence appeared in 2006 and involved cases from Italy, Germany, and Turkey (14–19). Many cases of PGB abuse have been reported by the Scandinavian, British, French, and German pharmacovigilance systems since 2008, most of which described patients currently or formerly dependent on other such substances (7).

PGB abuse was first mentioned in reports from the European Monitoring Centre for Drugs and Drug Addiction (EMCDDA) in 2010 (20). In November 2018, PGB was added to the list of psychoactive substances reviewed by the WHO 41^st^ Expert Committee on Drug Dependence (ECDD) (7) because of its liability for abuse and dependence and its potential to cause public health and social harm. PGB was classified as having considerable “abuse liability.” Over the past 10 years, PGB migrated from being a prescription drug to a recreational drug. It gradually became more accessible *via* online sources or on the black market (19, 21–28). PGB is often taken in combination with alcohol, BZDs, zopiclone, and gabapentin in an attempt to enhance the overall psychogenic effect. It has also been combined with cannabis, heroin, opioids, amphetamines, LSD, and mephedrone (19). The simultaneous use of PGB and opiates may have a potentially life-threatening interaction, and is associated with a significant increase in mortality (7). The abuse potential of PGB also has created concerns about increased risks of respiratory depression and death when used with opioids or other central nervous system depressants (29).

Individuals with prior or current drug abuse described pleasant PGB-related experiences attained at doses far exceeding those recommended by prescription (28). One thing common to several addictive drugs is that they increase the extracellular dopaminergic activity in the mesolimbic reward system. Different/unclear involvement of neurotransmitters and activation intensity of their receptors in cases of high/very high PGB dosage ingestion could justify the pleasant stimulation and euphoria described by users (28). PGB indeed was considered by users as an “ideal psychotropic drug” for recreational purposes, with effects similar to that of alcohol/GHB/BZDs such as entactogenic feelings and dextromethorphan-like disassociation (28).

The faster onset of a euphoric high and the linear relationship between blood concentrations and oral intake can be another reason why PGB is preferably self-administered by patients with experiences in substance abuse, such as opioid addicts (29).

PGB has been consumed concurrently with methadone to achieve an additional “high” (30). The prevalence of PGB abuse is up to 68% among those who abuse opioids (31). The reasons for this apparent association between opioid and PGB abuse remain to be elucidated. It has been suggested that since PGB is easily accessible, it can be an alternative drug choice when opioids or other drugs of such kind are difficult to obtain and because PGB produces only few adverse effects. Some studies have attributed the relatively higher rates of PGB misuse among opioid users to pain mismanagement and opioid withdrawal symptoms (14, 32, 33). Overall, a similar pattern is seen with other medications (e.g. venlafaxine, bupropion, quetiapine, and loperamide). PGB may induce a “liking” (euphoric high) subjective feeling, due to its GABA-mimetic action, with limited levels of “wanting”/behavioral dependence (28). On this basis, it can be hypothesized that PGB belongs to those substances with no relevant addictive power (“wanting”) but could become addictive in patients with prior substance abuse experiences (29). These patients look for various options to induce euphoria and to improve their affected stress reactivity that occurs following substance addiction (29).

PGB induces psychoactive effects including sedation, dissociation, relaxation, pleasure, drowsiness, disinhibition, improved sociability, empathy, and auditory and visual hallucinations (7). In a study on recreational sedative or hypnotic drug users, the subjective ratings provided by study participants who had taken 450 mg PGB resembled those given by participants who had consumed 30 mg diazepam (14, 34). High PGB abuse rates have been observed in patients with co-occurrences of psychoactive substance use and psychiatric disorders (dual diagnosis) (35, 36). This finding aligns with the observation that opioid addiction is common among patients with mental health disorders. Drug interactions, side effects, medication diversion, and substance abuse are very common in this vulnerable subpopulation (31). The aforementioned discoveries may have important implications in clinical practice (37).

### Rationale

To the best of our knowledge, this is the first study to furnish evidence of PGB abuse *via* hair analyses on a large cohort of 250 patients with a history of opiate dependency. As all individuals were under methadone maintenance therapy (MTT), they were being regularly monitored for abstinence from heroin, cocaine, cannabinoids, and non-prescribed BZDs. However, certain patients enrolled in these replacement programs may consume psychoactive substances not routinely or readily detected by toxicological analyses. As there has been a growing concern about PGB use within the MTT population, this drug was added to the target analyte panel. The aims of this study were to assess the extent of PGB misuse in the at-risk MTT population and to determine whether PGB is combined with one or more of the traditional drugs tested here. The study also investigated whether PGB abuse is correlated with specific daily methadone dosages, MTT duration, and psychiatry comorbidities. Hair analysis was the selected modality as it provided long-term information on drug use. Hair grows at an average rate of 1 cm mo^−1^ and maintains a chemical record of past drug consumption (38). Moreover, hair sampling is simple, rapid, minimally invasive, and supports the identification and quantitation of multiple analyses per sample (39).

## Methods

### Patients and Sample Collection

Laboratory procedures were conducted in accordance with the Helsinki Declaration of 1975 (revised 1983) and approved by the Bioethics Review Board of the University of Perugia (Protocol 2012‐006R). All participants provided informed consent. The study material consisted of 3‐cm hair samples collected from an area close to the scalp near the posterior vertex. The cohort consisted of 250 patients (age 18–60 y; 190 males; 60 females) under MTT for heroin addiction and receiving methadone doses ranging from 15–120 mg d^−1^. The patients were regularly monitored for drug consumption *via* hair analysis. All patients submitted lists of their diagnosed conditions and prescription medications and denied the use of any drugs outside the treatment plan.

### Reagents and Chemicals

Analytical grade solvents were purchased from Merck (Milan, Italy). Reagents, analytical drug standards, and their deuterated analogs were obtained from Sigma-Aldrich Corp. (Milan, Italy).

### Preparation and Analysis of Hair Samples

All hair samples were prepared, extracted, and derivatized by a fully validated, previously described method (38).

Opiates (codeine, 6-acetylmorphine, morphine), cocaine (and its metabolite benzoylecognine), cannabinoids (Δ9-*trans*-tetrahydrocannabinol and its metabolite 11-nor-9-carboxy-Δ9-tetrahydrocannabinol) and BZDs (alprazolam, clonazepam, delorazepam, diazepam, flurazepam, lorazepam, midazolam, nitrazepam, oxazepam, temazepam, and triazolam) were quantitated by gas chromatography-mass spectrometry (GC-MS; Focus gas chromatograph coupled with an ISQ; Thermo Electron Corp., Milan, Italy) in selective ion monitoring mode.

PGB was analyzed by gas chromatography/tandem mass spectrometry (GC-MS/MS; CP3800 gas chromatograph coupled with a Saturn 2000 ion trap; Varian, Harbor City, CA, USA) in multiple reaction monitoring (MRM) mode according to a previously described in-house procedure (38).

### Statistical Analysis

Descriptive statistics were calculated including frequencies, percentages, frequency tables for categorical variables, and means ± standard deviation (SD) for quantitative variables. Categorical variables were evaluated by χ^2^ or Fisher's exact test. Significance was set to ≤0.05 level in all tests.

Statistical analyses were performed with STATA v. 14.2 (StataCorp LP, College Station, TX, USA).

### Main Characteristics of the Cohort

Hair analysis investigated PGB consumption in 250 patients (190 males; 60 females; age range: 18–60 y) with a history of opiate dependency. All participants were treated with methadone at doses ranging from 15–120 mg d^−1^. The dosage groups were: a) low (LDG; ≤50 mg d^−1^; 170 patients; 68.0%); b) medium (MDG; 58 patients; 23.2%); and c) high (HDG; ≥100 mg d^−1^; 22 patients; 8.8%). Most patients had been receiving methadone for >3 y and were <50 y. The age distribution was as follows: 102 patients (40.80%; <30 y), 98 patients (39.20%; between ages 30 and 50 y), and 50 patients (20.0%; >50 y). BZDs were regularly prescribed to 33 patients (13.2%) for insomnia or general anxiety. PGB was regularly prescribed to 17 patients (6.8%) for chronic pain or general anxiety (**Table 1**).

**Table 1:** Main characteristics of 250 patients under methadone maintenance therapy (MTT).

Gender	<30 y (n = 102)	30–50 y (n = 98)	>50 y (n = 50)	Duration in MTT (<1 y)	Duration in MTT (3–5 y)	Duration in MTT (>5 y)	Low dose group (LDG) (n = 170)	Medium dose group (MDG) (n = 58)	High dose group (HDG) (n = 22)	Prescribed BZDs (n = 33)	Prescribed PGB (n = 17)
Males (190)	72 (70.59%)	71 (72.45%)	47 (94%)	29 (15.26%)	38 (20.0%)	123 (64.74%)	133 (70.0%)	45 (23.68%)	12 (6.32%)	24 (72.73%)	12 (70.59%)
Females (60)	30 (29.41%)	27 (27.55%)	3 (6%)	12 (20%)	17 (28.33	31 (51.67%)	37 (61.67%)	13 (21.67%)	10 (16.67%)	9 (27.27%)	5 (29.41%)

## Results

### Pregabalin and Other Drugs

Opiates were detected in the form of heroin metabolites (codeine, morphine, and 6-monoacetylmorphine) in 54/250 patients (21.60%). Cannabis (Δ⁹-*trans*-tetrahydrocannabinol and 11-nor-9-carboxy-Δ⁹-tetrahydrocannabinol) was present in 74/250 patients (29.60%). Cocaine (as the parent drug and its main metabolite benzoylecgonine) was found in 21/250 patients (8.40%). Methadone was identified in 250/250 patients (100%) as expected. There were no significant associations between the use of these drugs and methadone dosage **Supplementary Figure 1**.

Significant concomitant use of prescribed BZDs and opiates (*P* < 0.05) emerged in the study cohort. BZD misuse was detected in 49/250 patients (19.60%), but it was not significantly associated with age or methadone dosage. BZD use with and without prescription was significantly associated with cannabis consumption (*P* < 0.05). Non-prescribed PGB was detected in the hair of 35/250 patients (14%) (**Table 2**).

**Table 2:** Concomitant drug use in the methadone maintenance therapy (MTT) group.

	MTT group (N = 250)	P value
Opiates + cocaine	8	0.055
Opiates + cannabis	12	0.180
Opiates + BZDs with prescription	12	0.027
Opiates + BZDs without prescription	11	0.872
Cocaine + cannabis	3	0.108
Cocaine + BZDs without prescription	1	0.073
Cannabis + BZDs with prescription	15	0.032
Cannabis + BZD without prescription	24	0.001

Most of the PGB abusers were male (91.43%) and <50 y (48.57% < 30 y; 45.71% between ages 30 and 50 y). There were no apparent associations between PGB abuse and methadone dosage as 54.29% of the PGB abusers were in LDG, 31.43% were in MDG, and 14.29% were in HDG. There was no apparent association between PGB abuse and MTT duration (14.29% ≤1 y; 14.29% 1–3 y; 71.43% ≥3 y (**Table 3**).

**Table 3:** Main characteristics of PGB positive patients.

	Females	Males	<30 y	30–50 y	>50 y	Low methadone dose group (LDG)	Medium methadone dose group (MDG)	High methadone dose group (HDG)	Duration in MTT <3 y	Duration in MTT 3–5 y	Duration in MTT >5 y
**Unprescribed PGB** **(n = 35**)	3 (8.57%)	32 (91.43%)	17 (48.57%)	16 (45.71%)	2 (5.71%)	19 (54.29%)	11 (31.43%)	5 (14.29%)	5 (14.29%)	5 (14.29%9	25 (71.43%)

More than half (57.14%) of the PGB users concurrently used other drugs. In 11/20 cases, the second drug category was opiates. There were no significant differences in the combined use of PGB with opiates, cocaine, cannabis, or non-prescription BZDs (**Table 4**).

**Table 4:** Non prescribed PGB and combined drugs, as found in hair samples from the methadone maintenance therapy (MTT) group.

	MTT group (N = 250)
Unprescribed PGB total findings	35
PGB alone	15
PGB + opioids	11
PGB + cocaine	2
PGB + cannabis	4
PGB + BZDs with prescription	0
PGB + BZDs without prescription	3

### Psychiatric Comorbidity and PGB Abuse

Of the 250 MTT patients, 66 (26.40%) were diagnosed with a psychiatric condition. Depressive disorders (MDD, 36.36%) and GAD (33.33%) were the most commonly diagnosed psychiatric conditions followed by borderline personality (BDP, 21.2%), binge-eating disorder (BED, 4.55%), posttraumatic stress disorder (PTSD, 3.03%), and ADHD (1.52%). Of the 35 patients who abused PGB, 9 had psychiatric comorbidities (25.71%) including GAD (55.56%), MDD (33.33%), and BDP (11.11%) (**Table 5**).

**Table 5:** Psychiatric comorbidity and PGB abuse in the cohort.

Psychiatric comorbidity	Total findings	Positivity to unprescribed PGB
**ADHD**	1 (1.52%)	0
**BDP**	14 (21.21%)	1 (11.11%)
**BED**	3 (4.55%)	0
**GAD**	22 (33.33%)	5 (55.56%)
**MDD**	24 (36.36%)	3 (33.33%)
**PTSD**	2 (3.03%)	0

## Discussion

### Polydrug Abuse in the Cohort

Poly-drug use is extremely common among opiate-dependent individuals. Hair sample analysis is a unique and efficacious method of assessing the consumption of illicit or abused prescription drugs. A longitudinal analysis of 3-cm hair samples provided a retrospective timeline of MTT patient contact with various substances. A hair growth rate of 1 cm mo^−1^ was considered. The hair samples were obtained from 250 patients. Of these, 76% were males and the age range was 18–50 y. These patients were being administered methadone at doses ranging from a minimum of 15 mg d^−1^ to a maximum of 120 mg d^−1^. The duration of therapy ranged from 1 mo to 14 y.

Cannabis (29.60%) and heroin (21.60%) were the most heavily consumed illicit drugs whereas cocaine intake was comparatively low (8.40%). In agreement with previous studies on illicit drug use in opiate-dependent individuals, cannabis was consistently found to be the most commonly consumed drug in this population (40, 41) mainly because it was generally perceived as harmless and was less stigmatized than other recreational drugs. It was also readily available and easily accessible (40). However, the consumption of these drugs was not significantly correlated with methadone dosage.

With regard to prescription drugs, BZDs were included in the therapeutic programs of 13.2% of the MTT patients. However, BZDs misuse was widespread and 19.60% of the patients in the methadone program tested positive for non-prescribed BZDs. High-risk opioid users typically misuse BZDs to self-medicate and/or to potentiate the effects of the opioids (42). Several individuals within this sample group displayed significant concurrent use of opiates with prescribed BZDs and cannabis with prescribed and non-prescribed BZDs (*P* < 0.05).

### Pregabalin Abuse in the Cohort

There are growing concerns about the potential for PGB misuse particularly among patients with past or present substance abuse disorders (37). Thus, the determination of PGB abuse may be useful for drug addiction specialists. For this reason, PGB was added to the panel of drugs to be detected in the hair samples collected from the MTT patients. Pregabalin is prescribed for chronic pain and general anxiety. It was detected in 6.8% of the MTT patients. Non-prescribed PGB was found in 14% of the MTT patients. The data on PGB obtained here aligned with those of other studies conducted on patients with past or present histories of misuse or abuse of other substances (30, 32, 38, 43–46). A survey of MTT and buprenorphine patients in Georgia (47) revealed PGB misuse in 8.2% of the 506 participants. An anonymous voluntary survey run in six substance abuse clinics in Scotland reported 29/129 respondents (22%) abused PGB. All 29 were MTT patients (30). A Finnish study analyzed 200 urine samples from 82 methadone and buprenorphine/naloxone patients and reported PGB in 4% of the cases (48). Another Finnish study (44) tested 101 urine samples from 68 anonymous substance abuse patients detected PGB in 9% of the subjects from an opiate withdrawal facility and in 31% of the patients in a harm reduction facility. In Germany, 12.1% of 124 patients undergoing methadone or buprenorphine therapy were PGB-positive (15). In Ireland, 9.2% of 440 MTT patients were PGB-positive and the misuse rate was 7% (45). In Israel, a cross-sectional study comprising the urinalyses of 300 MTT patients detected PGB in 17.7% of the cases (46).

In the present study, 35 identified users of non-prescription PGB were predominantly male (91.43%). Of these 35 PGB consumers, 48.57% were <30 y and 45.71% were in the 30–50 y range. These findings corroborate those of previous studies stating that males and younger participants were more likely to use PGB possibly because of a relative lack of awareness of the inherent risks and/or a desire for a new experience (33, 37). No significant association was established between PGB use and methadone dose and duration in the MTT group. However, PGB intake tended to be comparatively lower in subjects receiving methadone doses >100 mg d^−1^. This finding is consistent with a report proposing that PGB is used to potentiate the effects of methadone (30).

In our cohort, PGB was frequently combined with other drugs (57.14%). The latter were mostly opiates (55%). This observation corroborates those of earlier reports (29, 36, 48). PGB has been used to alleviate opioid withdrawal symptoms and increase the psychotropic effects of other substances, especially opioids (37).

There is anecdotal evidence that PGB is sometimes taken to interrupt the prolonged ‘kicks’ associated with stimulants. Rarely, it is consumed solely for ‘kicks’ (38). Chronic pain is highly prevalent among MTT patients, so they might consume PGB for its analgesic efficacy. This factor may also account for the high PGB misuse rate among MTT patients (46). In our cohort, 26.4% of the patients had a psychiatric comorbidity. Of these, 69.7% presented with anxiety and depressive disorders. The use of illicit substances is widespread within this subpopulation (46). Only 9/250 of the patients (3.6%) who abused PGB also had psychiatric comorbidities. However, 9/35 patients abusing PGB also had psychiatric comorbidities (25.71%). This substantial rate was similar to that reported for the Israel study (46). Patients were diagnosed with GAD (55.56%), MDD (33.33%), and BDP (1.11%). These conditions often co-occur with substance abuse (46). The MTT patients may have taken PGB not only for recreational purposes but also as a form of self-medication to relieve anxiety, provide temporary mood enhancement, and manage the side effects of other drugs. Individuals suffering from mood or anxiety disorders or drug withdrawal symptoms may crave the effects of PGB, which perpetuates a substance abuse pattern in the attempt to mitigate adverse reactions to other medication.

The risk of death from PGB overdose is apparently low (49, 50). Conversely, the combination of PGB and opioids may have a strong depressant effect on the CNS. The addition of BZDs, alcohol, and/or cannabis may further exacerbate this problem (30).

PGB was implicated in 1.1% of all drug-related deaths in Scotland in 2010 (30). In a Finnish forensic study, 48.1% of all PGB-positive deaths were associated with concurrent opioid use (50).

The pharmacokinetic profile of PGB limits its potential for interaction with opioids. PGB is not metabolized and does not exhibit plasma protein binding. *In vitro* studies showed that PGB does not inhibit drug-metabolizing enzymes (7). Nevertheless, certain reports suggested that there is are interactions between PGB and clozapine and opioids (7). The combination of PGB and opioids attenuates the development of tolerance and physical dependence. Moreover, it may enhanced analgesia/opioid sparing effects (7). Potential pharmacodynamic interactions between PGB and opioids include impaired cognition and gross motor function. PCB combined with other CNS depressants such as ethanol and lorazepam may induce respiratory failure and coma (7).

PGB users generally procured the drug through their healthcare providers, family, acquaintances, internet purchase, legitimate prescriptions, and at foreign destinations (51). PGB was mainly orally self-administered but other routes such as injection, smoking, or inhalation were also reported (51). The patients examined in the present study did not furnish details on PGB acquisition or intake. Their referring sociosanitary practitioners claimed that the PGB was procured mainly through illicit online sources or street dealers.

Very high maximum daily doses (≤7,500 mg d^−1^; ≤ 3–20× in excess of the normal therapeutic range) were described in case and online anecdotal reports of PGB abuse (19), (25). The effects included sedation, dissociation, relaxation, pleasure, drowsiness, disinhibition, improved sociability, empathy, and auditory and visual hallucinations (7). Euphoria was reported in 1%–10% of all cases (52). This side effect could also lead certain patients to misuse large doses of PGB (51). Furthermore, PGB is readily accessible, inexpensive, and difficult to detect in routine toxicological tests.

### Legal Status of Pregabalin

As awareness of the potential harm associated with PGB has increased, certain countries have attempted to reduce risk by restrictive legislation. In the United States, the Drug Enforcement Administration (DEA) placed PGB and all products containing pregabalin into Schedule V of the Controlled Substances Act (7). In the United Kingdom, PGB and gabapentin were reclassified as Class C controlled substances in April 2019. Drugs in this category require a physical prescriber signature for each prescription rather than authorization by electronic prescriptions. In addition, the prescriptions expire after 28 d (53). These provisions emphasize the need for programmatic pharmacovigilance to manage suspected PGB abuse in patients with a history of substance misuse (particularly opioids) and comorbid psychiatric conditions.

### Utility of Hair Analysis in Clinical Pregabalin Abuse Monitoring

In view of the evidence for PGB misuse in the present and earlier studies, efforts should be made to raise awareness of the high risk of PGB abuse and predisposing risk factors in certain patients.

Unfortunately, most routine toxicological analyses do not include PGB determination. Consequently, its consumption rate in a susceptible population cannot be estimated and those patients actually using PGB without prescription and in combination with other drugs cannot be identified. Moreover, the lack of a modality for PGB detection in toxicological screening may enable patients to continue PGB abuse. Hair analysis could be useful in clinical practice in that it could help correlate suspicious behaviors or psychiatric symptoms such as euphoria, dissociation, and the onset of suicidal ideation with chronic PGB abuse. Hair analysis data could provide objective evidence for drug intake and support clinical management decisions. Furthermore, hair analysis can be used to assess the compliance of patients prescribed PGB to treat conditions for which it is indicated. In cases of off-label PGB use to manage bipolar disorder, alcohol/narcotic withdrawal states, and ADHD, there may be a relatively greater risk of misuse. Thus, careful monitoring is warranted as it is for other commonly abused drugs. Hair analysis is very reliable for the clinical monitoring of methadone compliance and PGB surveillance. It facilitates a much wider scope of retrospective toxicological investigation than standard blood or urinalyses (54). Most prior studies on PGB abuse were anecdotal or based on single urinalysis. When the results of hair analysis and urinalysis are compared, however, the former has a much higher diagnostic sensitivity for repeated drug use (55).

### Limitations of the Study

Concerns have been raised regarding the utility of hair analysis in drug compliance monitoring as there is an apparent lack of correlation between drug dosage/intake and the relative concentrations of the parent compounds and their metabolites in the hair. This lack of association may be explained by the difficulty in obtaining accurate retrospective data on drug consumption, unknown degrees of compliance, and variability in hair pigmentation among different subjects (56). For these reasons, hair samples may be taken monthly using the first 1-cm segment. Changes in drug dosage may be monitored and quantified by measuring the changes in the parent drug and/or metabolite concentrations in the hair segments (56). In this type of analysis, the patient being evaluated is also the control as the drug concentrations in the hair segments are compared against those collected from other segments of the same hair sample or against another sample taken from the same individual. In this manner, several sources of inter-individual variation such as pigmentation, metabolic capacity, and hair growth rate may be eliminated (56). Another limitation of the study is that no information could be gathered on the non-prescribed PGB sources or dosages. Moreover, we were unable to gain access to medical records, examine the accuracy of each psychiatric diagnosis, or establish the efficacy of each treatment. Filling in these information gaps and performing psychometric tests to enhance psychiatric evaluations could further improve PGB abuse assessments.

## Data Availability Statement

The datasets used and/or analyzed during the current study are available from the corresponding author on reasonable request.

## Ethics Statement

Laboratory procedures were in accordance with the Helsinki Declaration of 1975 as revised in 1983 and approved by Bioethics Review Board of University of Perugia (Protocol 2012-006R). All subjects who participated in the study provided their informed consent.

## Author Contributions

ML carried out hair samples collection. AnG and KA carried out sample preparation and hair analysis. MB verified the analytical methods. AlG analyzed the data. CG supervised the project and wrote the manuscript with input from all authors. All authors read and approved the final manuscript.

## Conflict of Interest

The authors declare that the research was conducted in the absence of any commercial or financial relationships that could be construed as a potential conflict of interest.

## Supplementary Material

The Supplementary Material for this article can be found online at: https://www.frontiersin.org/articles/10.3389/fpsyt.2019.01022/full#supplementary-material

Supplementary Figure 1 Levels of methadone, opiates, cocaine, cannabis, BDZs and PGB without prescription in the hair of methadone maintenance therapy (MTT) patients.
