# Supplementary figures and images for: Pregabalin Abuse in Combination With Other Drugs: Monitoring Among Methadone Patients
Source: Front Psychiatry. 2020 Feb 11;10:1022. doi: 10.3389/fpsyt.2019.01022 (PMC7026508; doi:10.3389/fpsyt.2019.01022)

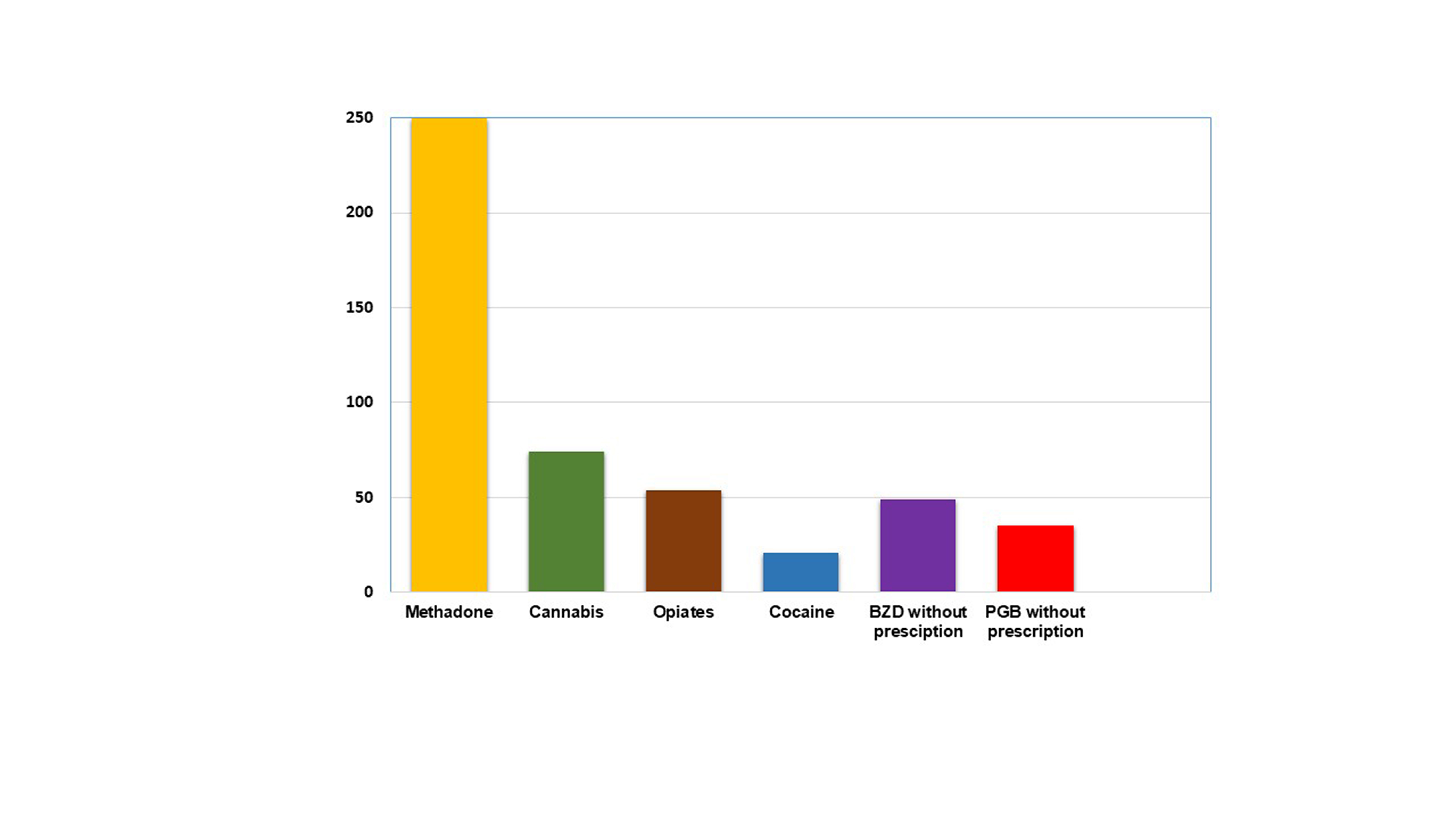

Supplement: Supplementary Figure 1 — Levels of methadone, opiates, cocaine, cannabis, BDZs and PGB without prescription in the hair of methadone maintenance therapy (MTT) patients. [file Image_1.jpeg]
